# Passenger Lymphocyte Syndrome Triggered Hemolytic Anemia Following Orthotopic Liver Transplantation: A Case Report

**DOI:** 10.1155/crh/8890213

**Published:** 2026-08-02

**Authors:** Teja Sureddi, Mariam Sharobeem, Monica Patel, Vik Bathula

**Affiliations:** ^1^ Virtua Health–Our Lady of Lourdes Hospital, Camden, New Jersey, USA; ^2^ Rowan-Virtua School of Osteopathic Medicine, Stratford, New Jersey, USA; ^3^ Hospital Medicine, Rowan-Virtua School of Osteopathic Medicine, Stratford, New Jersey, USA; ^4^ Department of Medicine, Rowan-Virtua School of Osteopathic Medicine, Stratford, New Jersey, USA, duhs.edu.pk; ^5^ Internal Medicine Residency, Chair, Virtua Our Lady of Lourdes Hospital, Camden, New Jersey, USA; ^6^ Department of Medicine, VMG, Virtua Our Lady of Lourdes Hospital, Camden, New Jersey, USA, duhs.edu.pk

## Abstract

Passenger lymphocyte syndrome (PLS) is a rare but recognized cause of immune‐mediated hemolytic anemia in solid organ transplant recipients, most often occurring in the setting of minor ABO incompatibility but also described with non‐ABO red cell antibodies. We present the case of a 48‐year‐old male with alcohol‐related cirrhosis who developed PLS following orthotopic liver transplantation. His postoperative hospital course was complicated by acute kidney injury, delirium, and severe anemia requiring intensive care unit readmission. Diagnostic workup demonstrated hemolysis with an acute hemoglobin drop, elevated lactate dehydrogenase, indirect hyperbilirubinemia, undetectable haptoglobin, a positive direct antiglobulin test, and anti‐B antibodies in the recipient serum. Management included donor‐type packed red blood cell transfusions, supportive care, close hemolysis monitoring, and adjustment of immunosuppressive therapy. This case underscores the importance of early recognition of PLS in postliver transplant patients with an acute hemoglobin decline and highlights the need for multidisciplinary coordination among transplant surgery, hematology, and transfusion medicine teams.

## 1. Introduction

Passenger lymphocyte syndrome (PLS) is a form of immune‐mediated hemolysis caused by the transfer of donor‐derived B lymphocytes into the recipient following solid organ transplantation. These donor lymphocytes may transiently produce antibodies directed against recipient red blood cell antigens, most commonly in the setting of minor ABO incompatibility, although PLS has also been described with non‐ABO red cell antibodies [1–3].

PLS is uncommon in the overall liver transplant population, but it is a recognized complication in minor ABO‐mismatched liver transplantation. Published reports describe PLS in approximately 30%–40% of minor ABO‐mismatched liver transplants, although only a subset of patients develop clinically significant hemolysis requiring intervention [1]. The typical onset occurs within 5–14 days after transplantation, with clinical features including an acute drop in hemoglobin, indirect hyperbilirubinemia, elevated lactate dehydrogenase, undetectable haptoglobin, and a positive direct antiglobulin test [4–6].

Given the complexity of the early postoperative liver transplant period, anemia may initially be attributed to blood loss, dilutional effects, infection, medications, or surgical complications. PLS should therefore be considered in postliver transplant patients who develop an acute hemoglobin decline without an identifiable bleeding source, particularly in the setting of minor ABO incompatibility.

### 1.1. Patient Information

A 48‐year‐old male with a past medical history of alcohol‐related cirrhosis, posttraumatic epilepsy not on active antiseizure medications, anxiety, chronic neck pain, jaundice, and chronic liver disease underwent orthotopic liver transplantation. The transplant was notable for a minor ABO mismatch; the donor blood type was O positive and hepatitis C antibody positive but nucleic acid testing negative. Anti‐B antibodies were later identified in the recipient serum, supporting donor‐derived antibody‐mediated hemolysis.

During transplantation and the immediate postoperative period, the patient received approximately 10 total units of blood products, including packed red blood cells and cryoprecipitate, for perioperative anemia and coagulopathy.

### 1.2. Transplant Procedure

The transplant was performed using the piggyback technique with duct‐to‐duct biliary anastomosis without stent placement, portosystemic bypass, and hepatic artery anastomosis using an aortic patch. Basiliximab and steroid induction were followed by maintenance immunosuppression with tacrolimus and mycophenolate mofetil.

### 1.3. Postoperative Course

The initial postoperative recovery was complicated by acute kidney injury, volume overload, and delirium on postoperative Day 4. The patient was extubated on postoperative Day 2, and his hemodynamics subsequently stabilized. He was transferred to acute rehabilitation on postoperative Day 9.

### 1.4. Clinical Deterioration and ICU Readmission

On postoperative Day 10, the patient was readmitted to the intensive care unit due to tachycardia, hypoxemia, and severe anemia, with hemoglobin decreasing from 7.8 g/dL to 3.4 g/dL without an identifiable source of bleeding. Imaging showed a stable chronic perihepatic fluid collection. Magnetic resonance cholangiopancreatography and ultrasound did not demonstrate active bleeding or biliary obstruction.

### 1.5. Diagnostic Workup

Hemolysis evaluation demonstrated elevated lactate dehydrogenase, rising indirect bilirubin, undetectable haptoglobin, and a positive direct antiglobulin test. Given the timing approximately 10 days after transplantation, absence of an overt bleeding source, minor ABO mismatch, and detection of anti‐B antibodies in the recipient serum, the findings were consistent with PLS.

### 1.6. Management

Management was primarily supportive and included transfusion with donor‐Type O positive packed red blood cells to avoid further antibody‐mediated hemolysis. Immunosuppression was adjusted by transitioning from tacrolimus to cyclosporine. The patient also received folate supplementation and frequent monitoring of hemoglobin and hemolysis markers. Corticosteroid escalation with prednisone 1 mg/kg was considered if clinical deterioration occurred.

### 1.7. Outcome

The patient’s hemoglobin stabilized between 7.7 and 8.0 g/dL, and lactate dehydrogenase and bilirubin levels trended downward. His mental status gradually improved with delirium precautions, melatonin, and quetiapine. The patient experienced a transient platelet decline, but heparin‐induced thrombocytopenia testing was negative. He remained afebrile, and no new infectious source was identified. A subhepatic fluid collection was drained by interventional radiology without purulent findings. He remained under intensive care observation with plans for transfer back to acute rehabilitation once clinically stable.

## 2. Discussion

PLS typically manifests within 1–3 weeks after transplantation and results from donor‐derived antibodies targeting recipient red blood cell antigens [4]. The patient developed hemolysis approximately 10 days after orthotopic liver transplantation, consistent with the reported time frame for PLS [5]. His laboratory findings, including elevated lactate dehydrogenase, indirect hyperbilirubinemia, undetectable haptoglobin, and a positive direct antiglobulin test, supported immune‐mediated hemolysis [5, 6]. The absence of overt bleeding and stable postoperative imaging further supported PLS as the cause of the acute hemoglobin decline.

Several risk factors for PLS have been described in the literature. These include minor ABO incompatibility, transplantation of lymphocyte‐rich grafts such as liver and lung, and persistence of viable donor‐derived B lymphocytes despite immunosuppressive therapy [1, 7]. PLS may also occur due to non‐ABO red cell antibodies, although ABO‐mediated cases remain the most frequently reported. The syndrome is often self‐limited as donor‐derived lymphocytes decline over time; however, clinically significant cases may result in profound anemia, repeated transfusion requirements, and intensive care monitoring [1–3].

Management is primarily supportive and includes transfusion of donor‐type red blood cells, as performed in this case with Type O positive packed red blood cells, to avoid further hemolysis [2]. Adjustment of immunosuppressive therapy may be considered to reduce donor‐derived B‐cell activity. Corticosteroids, intravenous immunoglobulin, rituximab, plasmapheresis, and red cell exchange have been described in severe or refractory cases, although evidence for these interventions remains limited to case reports and small case series [1, 5]. The role of rituximab remains incompletely defined, with variable outcomes reported in the literature.

Given that PLS typically develops within the first 1–3 weeks following transplantation, patients with known risk factors, particularly minor ABO‐mismatched liver transplantation, may benefit from anticipatory monitoring during the early postoperative period. This includes close follow‐up of serial hemoglobin levels and hemolysis markers such as lactate dehydrogenase, bilirubin, haptoglobin, and direct antiglobulin testing when clinically indicated. Early recognition can guide prompt donor‐compatible transfusion strategies, prevent unnecessary invasive investigations for bleeding, and support timely coordination among transplant surgery, hematology, and transfusion medicine teams.

## 3. Conclusion

PLS should be considered in liver transplant recipients who develop an acute drop in hemoglobin within the first three postoperative weeks, particularly in the absence of overt bleeding and in the setting of minor ABO incompatibility. Early recognition, donor‐type transfusion support, close monitoring of hemolysis markers, and individualized immunosuppression adjustment are central to management. This case highlights the importance of including PLS in the differential diagnosis of early posttransplant hemolytic anemia and underscores the value of multidisciplinary care.

## Funding

No funding was received for this manuscript.

## Conflicts of Interest

The authors declare no conflicts of interest.

## Supporting Information

Additional supporting information can be found online in the Supporting Information section.

## Supporting information


**Supporting Information** CARE Checklist for Case Reports.

## Data Availability

The data that support the findings of this study are available from the corresponding author upon reasonable request.
