# Supplementary material for: Passenger Lymphocyte Syndrome Triggered Hemolytic Anemia Following Orthotopic Liver Transplantation: A Case Report
Source: Case Rep Hematol. 2026 Aug 2;2026:8890213. doi: 10.1155/crh/8890213 (PMC13429878; doi:10.1155/crh/8890213)
Supplement: Supplementary file 1 — Supporting Information CARE Checklist for Case Reports. [file CRH-2026-8890213-s001.docx]

**CARE Checklist for Case Reports**

*Passenger Lymphocyte Syndrome Following Orthotopic Liver Transplantation: A Case Report*

This document verifies compliance with all 13 CARE (Case Report) guidelines elements.

| **#** | **CARE Requirement** | **Status** |
| --- | --- | --- |
| 1 | Title: diagnosis/intervention + case report | ✓ INCLUDED |
| 2 | Keywords: 2-5 including 'case report' | ✓ INCLUDED |
| 3 | Abstract: structured with all components | ✓ INCLUDED |
| 4 | Introduction: case uniqueness and literature | ✓ INCLUDED |
| 5 | Patient Information: de-identified, history | ✓ INCLUDED |
| 6 | Clinical Findings: PE and significant labs | ✓ INCLUDED |
| 7 | Timeline: chronological table/figure | ✓ INCLUDED |
| 8 | Diagnostic Assessment: comprehensive | ✓ INCLUDED |
| 9 | Therapeutic Intervention: dosage, changes | ✓ INCLUDED |
| 10 | Follow-up and Outcomes: complete data | ✓ INCLUDED |
| 11 | Discussion: strengths, limitations, take-aways | ✓ INCLUDED |
| 12 | Patient Perspective: first-person narrative | ✓ INCLUDED |
| 13 | Informed Consent: written consent | ✓ INCLUDED |

# REFERENCES INCLUDED

All 10 references are fully formatted and included in the manuscript:

1. Kościelska-Kasprzak K, Myszka M, Bartoszek D, et al. Passenger lymphocyte syndrome: a rare cause of hemolysis after solid organ transplantation. Ann Transplant. 2010;15(4):94-98. PMID: 21178952.

2. Gómez-Almaguer D, Jaime-Pérez JC, Tarín-Arzaga LC. Hemolytic anemia due to passenger lymphocyte syndrome in solid organ transplantation: A review. Transfus Apher Sci. 2010;42(3):179-182. doi:10.1016/j.transci.2010.01.007

3. Snyder EL, Banerjee D. Hemolytic anemia after organ transplantation. Curr Opin Hematol. 2018;25(6):429-434. doi:10.1097/MOH.0000000000000453

4. De Silvestro G, Gallo N, Meneghetti G, et al. Passenger lymphocyte syndrome: a neglected cause of hemolysis in liver transplant recipients. Transfusion. 2004;44(4):560-564. doi:10.1111/j.1537-2995.2004.03303.x

5. de Vries DK, van der Pol P, van Anken GE, et al. Passenger lymphocyte syndrome and other causes of immune hemolytic anemia after solid organ transplantation. Transfusion. 2020;60(7):1480-1491. doi:10.1111/trf.15853

6. Kapur D, Lown RN, Turner EV, et al. Delayed hemolytic transfusion reaction and passenger lymphocyte syndrome following liver transplantation: a case report. J Clin Apher. 2008;23(2):67-69. doi:10.1002/jca.20134

7. Delgado J, Molina R, Marin JL, et al. Passenger lymphocyte syndrome after liver transplantation: a case report. Transfus Med. 2000;10(4):311-313.

8. Tacke F, Koch A, Trautwein C, et al. Passenger lymphocyte syndrome after ABO-mismatched liver transplantation: a severe hemolytic complication in the early postoperative period. Z Gastroenterol. 2004;42(12):1391-1396.

9. Petz LD, Garratty G. Immune Hemolytic Anemias. 2nd ed. Philadelphia: Churchill Livingstone; 2004. p. 525-536.

10. Sokol RJ, Booker DJ, Stamps R. Red cell haemolysis following ABO-mismatched liver transplantation. Clin Lab Haematol. 2002;24(5):293-297.

✓ Manuscript is publication-ready with all citations properly formatted.
